# Ludens: A Gambling Addiction Prevention Program Based on the Principles of Ethical Gambling

**DOI:** 10.1007/s10899-021-10066-7

**Published:** 2021-08-23

**Authors:** Mariano Chóliz, Marta Marcos, Francisco Bueno

**Affiliations:** 1 Psychology School, University of Valencia, Avda. Blasco Ibáñez, 21, 46010 Valencia, Spain; 2 Addictions Service, Valencia City Council, Plaza del Ayuntamiento, 1, 46001 Valencia, Spain

**Keywords:** Gambling disorder, Prevention program, Addiction prevention, Adolescence, Ethical gambling

## Abstract

Gambling is legal in most countries. However, despite having some economic benefits, certain characteristics of gambling can have health consequences, rendering it a public health issue. The effects can be summarized according to the following three “laws” of ethical gambling: “Gambling Dynamics Law”: companies’ economic gains come directly from players’ losses; “Expected Loss Law”: the more one gambles, the greater the probability of losing; and “Addiction Law”: the more one gambles, the greater the need to play again, leading to further losses. Ludens is a gambling addiction prevention program that has four goals: inform participants about gambling and gambling addiction; sensitize participants to the risk of gambling for health, especially addiction; promote a change in attitudes toward gambling; and alert participants to risky behaviors that can lead to addiction. The prevention program was implemented during 2017 to 2019. Fourteen psychologists presented it to 2372 adolescents (48.8% females, 51.2% males) aged 14–19 years, none of whom were university students, recruited from 42 Spanish high schools in 132 groups taking different courses. The main dependent variables analyzed were the monthly frequencies of gambling, at-risk gambling, and gambling addiction (as measured by the National Opinion Research Center DSM-IV Screen for Gambling Problems, adapted to diagnose gambling disorder according to DSM-5, in which pathological gambling is considered an addictive disorder). Given that all of the gamblers were adolescents (most were minors), fulfilment of 1–3 the DSM-5 diagnostic criteria was considered to indicate a risk of problem gambling. After the administration of Ludens, statistically significant reductions were observed in the three variables of interest: monthly frequency of gambling, percentage of adolescents with risky gambling, and percentage of adolescents with gambling disorder. The results were analyzed according to sex and age (minors vs. adolescents between 18 and 19 years old). The results obtained after applying the prevention program indicate that Ludens is effective as a universal prevention program for gambling addiction.

## Introduction

Gambling disorder is a mental health problem. Initially it was considered an impulse control disorder (APA, 1980; WHO, 1977), although the incorporation of some characteristic criteria of substance dependence disorder in the DSM-III-R (APA, 1987), such as tolerance or withdrawal syndrome, led to pathological gambling being considered a disorder that should be included in the same category as drug addiction (Petry, 2006; Potenza, 2006).

Following expert committee discussions, the major current classifications of mental disorders (APA, 2013; WHO, 2021) now consider gambling disorder an addictive disorder, as it activates the same reward circuits as drugs and its clinical diagnostic criteria are similar to those for substance use disorders.

Gambling is an enormously lucrative activity for some companies and administrations (Markham & Young, 2015), which encourage the establishment of very favorable conditions for its expansion (Chóliz & Sáiz-Ruiz, 2016a) including large numbers of gambling halls, advertisements via all types of media (TV, Internet, public spaces, etc.), easy access to the most addictive forms of gambling (online slot machines, electronic gaming machines, etc.), and the use of effective marketing strategies to promote gambling (loyalty schemes, bonuses, etc.). These activities are typically supported by laws and regulations that are very favorable to the promotion of gambling as an economic activity. These conditions, combined with gambling’s addictive potential, have led to gambling disorder being considered a public health problem (Korn & Shaffer, 1999; Latvala et al., 2019; Wardle et al., 2019) as well as a mental disorder. Thus, it is important that prevention measures be undertaken.

The characteristics of gambling, and the conditions under which it occurs, can create a public health problem based on what we term here three “laws” of ethical gambling:

- First law (*“Gambling Dynamics Law”*): *“In gambling, money is neither created nor destroyed; it only travels from one pocket to another.”*
- Second law (*“Expected Loss Law”*): *“The probability of losing on the part of the gambler increases proportionally to the frequency with which money is gambled.”*
- Third law (*“Addiction Law”*): *“The more you gamble, the greater the need to gamble again, and this increases the probability of developing a mental disorder.”*

At this juncture, it is necessary to distinguish between responsible gambling and ethical gambling. According to the Reno Model (Blaszczynski et al., 2004), a responsible gambling program rests upon two main principles: the ultimate decision of whether to gamble resides with the individual, i.e., they have a choice; and when making the decision, individuals must have the opportunity to be informed. In contrast, the theory of ethical gambling (Chóliz, 2018) considers that information is insufficient to prevent gambling disorders in society because gambling is potentially addictive; the majority of the diagnostic criteria for gambling disorder reflect excessive gambling; and gambling companies encourage excessive gambling.

Gambling disorder is considered an addictive disorder because it activates the same reward circuits as drugs, such that there is a need to repeat the behavior (APA, 2013). Considering gambling as an addictive disorder has very relevant implications for its prevention. Regardless of differential vulnerability to the development of the disease, gambling disorder occurs because people engage in potentially addictive behavior under environmental conditions that favor the development and maintenance of addiction (Chóliz & Sáiz-Ruiz, 2016b). It is through these variables that the necessary preventive intervention can be undertaken.

The prevalence rates of gambling disorder have varied remarkably among studies (Calado et al., 2017). This may be due both to methodological issues (sampling procedures, use of different diagnostic tests) and to differences in the promotion of gambling in various societies or at different times. Other factors, such as environmental variables (availability, accessibility, etc.), are also relevant in influencing the incidence of gambling disorder and depend to a large extent on the regulatory measures established by governments.

One area of solid empirical evidence indicates that youth and adolescents exhibit higher prevalence rates of gambling addiction (Delfabbro et al., 2016; Gupta & Derevensky, 1998). This may be due both to the vulnerability of adolescents to the development of addictions (Chambers et al., 2003; Potenza, 2006) and to the wide promotion among young people of games, such as sports betting, as a form of leisure. Added to this greater vulnerability and promotion of gambling in youth is the fact that the games preferred by young people, such as online gambling, are also potentially the most addictive ones (Chóliz et al., 2019).

Despite the fact that gambling addiction is a public health problem that affects young people and adolescents, few studies in the scientific literature have evaluated the effectiveness of prevention programs. A systematic review of gambling addiction prevention programs in young people was carried out using the Preferred Reporting Items for Systematic Reviews and Meta-analyses (PRISMA) (González, 2020). Key words searched for in the scientific databases included “prevention”, “program”, “gambling” and “school”. The searches yielded 398 articles. The Population, Intervention, Comparison, Outcomes and Study (PICOD) framework was used to determine the eligibility of the articles. Studies were selected if they met the following criteria:

- Type of study: original research published in a scientific journal.
- Design: empirical study.
- Type of prevention: universal.
- Place of implementation: non-university educational establishments.
- Sample: adolescents.
- Analysis: quantitative pre–post intervention data.

Twenty-one articles were included in our review (Calado et al., 2019; Canale et al., 2016; Donati et al., 2014; Ferland et al., 2002; Huic et al., 2017; Ladouceur et al, 2003, 2004, 2005; Lemaire et al., 2004; Lupu & Lupu, 2013; Parham et al., 2019; Ren et al., 2019; St-Pierre et al., 2017; Taylor & Hillyard, 2009; Todirita & Lupu, 2013; Turner et al., 2008a, b; Walther et al., 2013; Williams, 2002, 2004; Williams et al., 2010). Of these studies, only seven used an evaluation procedure to classify participants who currently had or were at risk of having an addictive disorder (Calado et al., 2019; Canale et al., 2016; Donati et al., 2014; Ren et al., 2019; Turner et al., 2008a; Williams et al., 2002, 2010). The scales used were the South Oaks Gambling Screen-Revised for Adolescents (SOGS-RA), the Diagnostic and Manual of Mental Disorders-IV (DSM-IV), and the Canadian Problem Gambling Index (CPGI).

The efficacy assessment in many studies focused mainly on cognitive variables (erroneous beliefs and fallacies about gambling, concept of randomness, illusion of control, superstitious thinking, etc.). Many studies only evaluated cognitive variable (Donati et al, 2014; Ferland et al., 2002; Ladouceur et al., 2003, 2004, 2005; Lemaire et al., 2004; Lupu & Lupu, 2013; Parham et al., 2019; Ren et al., 2019; St-Pierre et al., 2017; Taylor & Hillyard, 2009; Todirita & Lupu, 2013; Turner et al., 2008a, b; Walther et al., 2013), despite the fact that changes therein are not usually correlated with a decrease in gambling behavior (Williams, West, & Simpson, 2012). In a few studies, the efficacy of prevention programs was evaluated based on objective indices such as a reduction in gambling behavior (Calado et al., 2019; Canale et al., 2016; Huic et al., 2017; Williams, 2002, 2004; Williams et al., 2010). Furthermore, many of these studies were based on small samples, making it difficult to assess the effectiveness of prevention programs for people who had problems with gambling or gambling disorder. Only four studies evaluated prevention programs involving than 1000 adolescents (Ren et al., 2019; Taylor & Hillyard, 2009; Walther et al., 2013; Williams et al., 2010).

The work we present here aims to evaluate the effectiveness of the Ludens prevention program using objective behavioral and clinical measures recorded before and after applying the prevention program.

### Procedure

Ludens (Chóliz, 2017) consists of two sessions, which take place in small groups. The sessions are directed by a psychologist specializing in addiction and gambling. All students agreed to participate in the prevention program. Additionally, parental consent was obtained for minors to participate in the prevention program and complete the questionnaires, which were completely anonymous.

All contents are supported by audiovisual resources, such as diagrams, graphics, news stories, testimonies of gamblers and patients, and advertisements illustrating the techniques used by companies to induce gambling and their main marketing techniques. Two very relevant points are explained in detail: the process by which addiction develops and the DSM-5 diagnostic criteria for gambling disorder. These criteria are illustrated with videos of real testimonies from people with gambling problems or gambling addiction, some of whom are well-known public figures. During the two sessions, a total of 28 video sequences are presented, each between 20 s and a minute and 30 s long. All of these are briefly explained and discussed.

The session contents are as follows:

- *Session* 1: Characteristics of gambling and games; economic dimension of gambling; the “*three laws of ethical gambling*” (economic and psychological laws); types of gamblers; gambling as a mental disorder.
- *Session* 2: Online gambling; addiction to online gambling, with a focus on structural and environmental characteristics; promotion of online gambling, particularly advertising and marketing; responsible gambling and corporate social responsibility; ethical gambling; risks of gambling; alerts and recommendations to prevent gambling addiction.

### Objectives

Ludens is based on the principles of ethical gambling (Chóliz, 2018), which aim to privilege citizens’ right to health over gambling companies’ economic benefits. The main objectives of the prevention program are the following:

1. Inform. Gambling is an economic activity of enormous magnitude (billions of euros annually), which is being widely promoted because it is very profitable for companies (Markham & Young, 2015). All types of gambling are designed so that the operator wins in the long run (Chóliz, 2018). But gambling is also a psychological activity that can lead to a mental disorder (APA, 2013). The diagnostic criteria for gambling disorder are explained.
2. Sensitize. Ludens sensitizes participants to the idea that gambling companies and some public administrators are interested in promoting gambling in society (Chóliz & Sáiz-Ruiz, 2016a). People should know the techniques used for promoting gambling and the risks that gambling poses for mental health (e.g., loyalty bonuses; a proliferation of gambling houses in cities, even in marginal neighborhoods; the presence of gambling machines in hospitality establishments; and presenting gambling as a risk-free leisure activity if people "gamble responsibly"). Importantly, although not all games have the same addictive capacity, none are without risk, and online gambling places an additional burden on those predisposed to gambling (Papineau et al, 2018).
3. Change attitudes. One of the main objectives of Ludens is for people to see gambling from another point of view. The companies’ profits come exclusively from gamblers’ losses (Latvala et al., 2019), and many entities involved in multinational companies are interested in obtaining gamblers' money.
  Many of the testimonials, advertisements, graphics, news, etc., that are presented in the Ludens sessions are intended to corroborate the three laws of ethical gambling.
4. Avoid risky behaviors. The behaviors that should be avoided with respect to gambling are identified, i.e., do not drink alcohol; do not gamble again to recover your losses; do not gamble to overcome boredom or emotional distress, etc.

It is important to note that this is not about promoting responsible gambling (Blaszczynski et al., 2004, 2011) or fostering safe gambling, but rather about avoiding forms of gambling that are most likely to lead to addiction. Ludens is especially critical of how gambling companies use the term "responsible gambling" to divert responsibility for the development of gambling disorder to the gambler (Cassidy, 2020; Hancock & Smith, 2017; Livingstone et al., 2014) and of the consequences that this belief has for a pathological gambler’s mental health.

The objective of the research presented in this article was to evaluate the efficacy of Ludens, a gambling addiction prevention program, for use in adolescents aged 14–19 years.

## Materials and Methods

### Participants

Ludens was implemented in 2372 adolescents (48.8% females and 51.2% males) aged 14–19 years.

### Procedure

Forty-two high schools in 19 Spanish cities and towns asked the University of Valencia to implement the Ludens program in their facilities. Sessions were presented to 132 groups by 14 different technicians over three academic years from 2017 to 2019.

First, a brief survey on the patterns of gambling (frequency with which one participates in each legal form of gambling in Spain) and gambling disorder was administered. A rating scale was used to evaluate the frequency of both traditional forms of gambling (lotteries, casinos, slot machines, etc.) and online gambling (bets, slots, roulette, etc.). Frequencies were rated using a six-point Likert scale ranging from "I have never played that game" to "I play it every day or almost every day." To assess problems associated with gambling, the National Opinion Research Center DSM-IV Screen for Gambling Problems (NODS) (Gernstein et al., 1999) was used.

These same variables were measured using the same procedure 1 month after Ludens was administered to observe changes resulting from the gambling addiction prevention program.

### Analysis

A ***test–retest analysis*** of the main variables involved in gambling addiction was carried out to evaluate the effectiveness of the gambling addiction prevention program.

The ***dependent variables*** were the monthly gambling frequency and the presence or absence of problems caused by gambling, i.e., gambling disorder and at-risk gambling.

- *Monthly gambling frequency* was considered an indication of regular dedication to gambling, which was defined as one to three times per month or more. Operationalizing the frequency of gambling was considered useful because it is relatively normal in Spain to engage at some time in one’s life in some games of chance, such as lotteries. Nevertheless, gambling occurring at a rate of one to three times a month implies that it has become an activity to which an individual dedicates time and resources. This is a very relevant consideration because minors are prohibited from gambling in Spain.
- *Gambling disorder*. NODS (Gernstein et al., 1999) was used to evaluate problems associated with gambling. A diagnosis of gambling disorder applies when four of the criteria listed in the DSM-5 are met (APA, 2013).
- *At-risk gambling*. Given that the participants were adolescents (mostly minors), it was considered that fulfillment of one to three of the nine DSM-5 criteria was sufficient to indicate a risk of problem gambling.

The ***independent variable*** was whether the Ludens sessions was administered (in a small group of 15–20 people).

Because the evaluation of the test–retest differences was carried out between dependent variables whose values were measured in percentages, chi-square tests were applied to contrast the hypotheses, and phi values were calculated to analyze the effect size.

In most analyses, sex (females vs. males) and age (minors vs. adolescents of legal age) were considered.

### Hypotheses

#### Main Hypothesis

- After the prevention program, the percentage of adolescents who gamble monthly will decrease significantly, as will the percentages of adolescents who exhibit behaviors corresponding to at-risk gambling and gambling disorder.

#### Secondary Hypotheses

- Before the application of Ludens, males gamble more frequently in games of chance than females and have higher rates of at-risk gambling and gambling disorder.
- Before the application of Ludens, adolescents aged 18–19 years gamble more frequently in games of chance than minors and have higher rates of at-risk gambling and gambling disorder.
- Sports betting has the highest monthly prevalence of any type of game. Males and adults will bet more than females and minors, respectively.

## Results

### Frequency of Gambling Before the Implementation of a Gambling Prevention Program

Table 1 shows the percentage of adolescents who engaged in each of the legal forms of gambling on a monthly basis.

**Table 1:** Percentage of adolescents who gamble monthly in each type of game

	Females (%)	Males (%)	Minors (%)	18–19 years (%)
Lotteries	2.33	3.17	2.52	4.27
Offline sport betting	1.09	4.58	2.84	3.16
Scratch cards	1.62	3.22	2.27	3.64
Slot machines	0.71	6.58	3.14	6.96
Casino games	0.95	8.03	3.11	12.97
Poker	0.33	2.40	1.38	1.58
Bingo	1.30	1.21	1.35	0.74
Online poker	0.81	3.13	1.98	2.06
Online bingo	0.38	0.82	0.62	0.47
Online casinos	0.57	2.63	1.52	2.22
Online slots	0.29	1.59	0.95	0.95
Online sport betting	1.81	18.00	9.28	14.87

With respect to sex differences, boys gambled more than girls in most games. Differences were statistically significant for offline sport betting (*X*^*2*^_*1*_ = 46.78, *p* < 0.001, *Phi* = 0.10), scratch cards (*X*^*2*^_*1*_ = 111.62, *p* < 0.001, *Phi* = 0.05), slot machines (*X*^*2*^_*1*_ = 103.40, *p* < 0.001, *Phi* = 0.16), casino games (*X*^*2*^_*1*_ = 123.44, *p* < 0.001, *Phi* = 0.17), poker (*X*^*2*^_*1*_ = 33.59, *p* < 0.001, *Phi* = 0.09), online poker (*X*^*2*^_*1*_ = 29.61, *p* < 0.001, *Phi* = 0.08), online casinos (*X*^*2*^_*1*_ = 28.55, *p* < 0.001, *Phi* = 0.08), online slots (*X*^*2*^_*1*_ = 19.34, *p* < 0.001, *Phi* = 0.07), and online sport betting (*X*^*2*^_*1*_ = 310.92, *p* < 0.001, *Phi* = 0.27). The effect size was very large in the case of online betting.

Adolescents aged 18–19 years gambled more than minors in lotteries (*X*^*2*^_*1*_ = 6.17, *p* < 0.001, *Phi* = 0.04), slot machines (*X*^*2*^_*1*_ = 22.16, *p* < 0.001, *Phi* = 0.07), casino games (*X*^*2*^_*1*_ = 120.85, *p* < 0.001, *Phi* = 0.17), and online sport betting (*X*^*2*^_*1*_ = 18.60, *p* < 0.001, *Phi* = 0.07). The effect size was very large in the case of casino games.

### Frequency of Gambling Before and After the Implementation of Ludens: *Sex Differences*

Table 2 shows the percentage of females and males who gambled monthly before and after the application of Ludens.

**Table 2:** Percentages of females and males who gambled monthly before and after the application of Ludens

	Traditional gambling		Online gambling	
	Before (%)	After (%)	Before (%)	After (%)
Males	63.5	46.8	48.0	35.5
Females	47.7	33.5	15.8	10.8
Total	55.6	40.4	32.2	23.5

Before the implementation of Ludens, boys gambled more frequently than girls in both traditional (*X*^*2*^_*1*_ = 59.63, *p* < 0.001, *Phi* = 0.16) and online forms of gambling (*X*^*2*^_*1*_ = 279.99, *p* < 0.001, *Phi* = 0.35).

After the implementation of Ludens, the percentages of adolescents who engaged monthly in traditional gambling (*X*^*2*^_*1*_ = 99.26, *p* < 0.001, *Phi* = 0.15) and online gambling (*X*^*2*^_*1*_ = 39.93, *p* < 0.001, *Phi* = 0.10) decreased significantly. Statistically significant reductions in the rates of traditional and online gambling were observed for both females (*X*^*2*^_*1*_ = 42.78, *p* < 0.001, *Phi* = 0.14; *X*^*2*^_*1*_ = 10.86, *p* < 0.001, *Phi* = 0.07, respectively) and males (*X*^*2*^_*1*_ = 61.51, *p* < 0.001, *Phi* = 0.17; *X*^*2*^_*1*_ = 34.86, *p* < 0.001, *Phi* = 0.13, respectively).

### Frequency of Gambling Before and After the Implementation of Ludens: *Age Differences*

Table 3 shows the percentages of minors and adolescents aged 18–19 years who gambled monthly before and after the application of Ludens.

**Table 3:** Percentages of minors and adolescents aged 18–19 years who gambled monthly before and after the application of Ludens

	Traditional gambling		Online gambling	
	Before (%)	After (%)	Before (%)	After (%)
Minors	53.9	37.8	31.1	22.1
18–19 years	66.2	55.4	39.2	31.5

Before the implementation of Ludens, adolescents aged 18–19 years gambled more frequently than minors using both traditional (*X*^*2*^_*1*_ = 17.43, *p* < 0.001, *Phi* = 0.09) and online forms of gambling (*X*^*2*^_*1*_ = 8.76, *p* < 0.01, *Phi* = 0.06).

After the implementation of Ludens, statistically significant reductions were observed in the percentages of minors who participated in both traditional and online gambling (*X*^*2*^_*1*_ = 96.03, *p* < 0.001, *Phi* = 0.16; *X*^*2*^_*1*_ = 37.42, *p* < 0.001, *Phi* = 0.10, respectively). The percentage of adolescents aged 18–19 years who participated in both traditional and online gambling also decreased significantly (*X*^*2*^_*1*_ = 7.73, *p* < 0.01, *Phi* = 0.11; *X*^*2*^_*1*_ = 4.05, *p* < 0.05, *Phi* = 0.08, respectively).

### At-risk Gambling and Gambling Disorder Before and After the Implementation of Ludens: *Sex Differences*

Table 4 shows the percentages of females and males exhibiting at-risk gambling behavior and diagnosed with gambling disorder before and after the application of Ludens.

**Table 4:** Percentages of females and males with gambling problems before and after the application of Ludens

	At-risk gambling		Gambling disorder	
	Before (%)	After (%)	Before (%)	After (%)
Males	22.1	12.3	3.4	1.7
Females	5.6	2.8	0.6	.4
Total	13.9	7.7	2.0	1.1

Before the implementation of Ludens, males exhibited behaviors corresponding to both at-risk gambling (*X*^*2*^_*1*_ = 132.75, *p* < 0.001, *Phi* = 0.24) and gambling disorder (*X*^*2*^_*1*_ = 23.46, *p* < 0.001, *Phi* = 0.10) more frequently than did females.

After the implementation of Ludens, the percentages of adolescents who participated in at-risk gambling (*X*^*2*^_*1*_ = 41.43, *p* < 0.001, *Phi* = 0.10) and exhibited gambling disorder (*X*^*2*^_*1*_ = 6.17, *p* < 0.01, *Phi* = 0.04) decreased significantly. Significantly smaller proportions of females (*X*^*2*^_*1*_ = 9.70, *p* < 0.01, *Phi* = 0.07) and males (*X*^*2*^_*1*_ = 35.11, *p* < 0.01, *Phi* = 0.13) engaged in at-risk gambling. A significantly smaller proportion of males was also diagnosed with gambling disorder (*X*^*2*^_*1*_ = 6.39, *p* < 0.01, *Phi* = 0.05).

### At-risk Gambling and Gambling Disorder Before and After the Implementation of Ludens: *Age Differences*

Table 5 shows the percentages of minors and adolescents aged 18–19 years exhibiting at-risk gambling behavior and diagnosed with gambling disorder before and after the application of Ludens.

**Table 5:** Percentages of minors and adolescents aged 18–19 years with gambling problems before and after the application of Ludens

	At-risk gambling		Gambling disorder	
	Before (%)	After	Before	After
Minors	12.6	6.6	1.9	.7
18–19 years	21.6	13.5	3.0	3.0

Before the implementation of Ludens, a greater proportion of adolescents aged 18–19 years participated in at-risk gambling compared to minors (*X*^*2*^_*1*_ = 18.94, *p* < 0.001, *Phi* = 0.09), but no age difference in gambling disorder was found.

After the implementation of Ludens, statistically significant reductions were observed in the percentages of minors who exhibited at-risk gambling behavior (*X*^*2*^_*1*_ = 36.17, *p* < 0.001, *Phi* = 0.10) and were diagnosed with gambling disorder (*X*^*2*^_*1*_ = 8.92, *p* < 0.01, *Phi* = 0.05). Statistically significant reductions were also observed for at-risk gambling (*X*^*2*^_*1*_ = 6.87, *p* < 0.01, *Phi* = 0.11), but not gambling disorder, among adolescents aged 18–19 years.

### Diagnostic Criteria Before and After the Implementation of Ludens

Table 6 shows the frequency with which gamblers (i.e., those who played some or several games before the application of Ludens) met each of the nine DSM-5 diagnostic criteria for gambling disorder.

**Table 6:** Percentages of gamblers who met each diagnostic criterion for gambling disorder before and after the application of Ludens

	Before (%)	After (%)
Preoccupation with gambling	17.2	13.2
Chasing losses	9.7	5.4
Escape	5.3	3.1
Absence of control	4.4	2.4
Personal problems	4.0	3.2
Tolerance	3.6	3.4
Withdrawal syndrome	3.1	2.5
Lying	2.7	2.0
Need help from others	1.3	.8

Frequencies before and after the application of Ludens are shown.

The most frequently met DSM-5 diagnostic criteria were “preoccupation with gambling” and “chasing losses.” The frequencies of meeting some diagnostic criteria decreased after the Ludens sessions, including those associated with “preoccupation with gambling” (*X*^*2*^_*1*_ = 6.57, *p* < 0.01, *Phi* = 0.05), “chasing losses” (*X*^*2*^_*1*_ = 14.03, *p* < 0.001, *Phi* = 0.08), “gambling as a form of escapism” (*X*^*2*^_*1*_ = 6.62, *p* < 0.01, *Phi* = 0.05), and “absence of control” (*X*^*2*^_*1*_ = 6.10, *p* < 0.01, *Phi* = 0.05).

Although this article does not involve qualitative analysis, the adolescents nevertheless made some interesting comments during the program, such as “*If I don’t bet, I don’t have fun watching football*.” They were surprised to learn that there is no strategy to “beat the casino” and that tipsters make money primarily from their fees, not their own bets.

## Discussion and Conclusions

Ludens (Chóliz, 2017) is a gambling addiction prevention program based on the principles of ethical gambling (Chóliz, 2018), which are summarized in the so-called three laws of ethical gambling. The entire program revolves around these three laws, which are illustrated with examples supported by extensive audiovisual materials. The prevention program has the following four objectives: (a) informing audiences about gambling and gambling addiction, (b) raising awareness about gambling disorder, (c) provoking a change in attitudes toward gambling, and (d) warning individuals about risky behaviors that can lead to addiction.

Despite the complexity and multidimensionality of gambling, the prevention program focuses on two dimensions whose special characteristics favor the development of gambling addiction in society: the economic and psychological dimensions.

Regarding the economic dimension, gambling is a legally authorized activity in many countries. It is carried out by companies and administrations whose monetary benefits are derived from players’ losses. Thus, it is, in terms of game theory (Morgenstern & Von Neumann, 1953), a zero-sum activity as defined by the first law, or the “Gambling Dynamics Law.”

The second point is that the rules of all games are designed so that, following the laws of probability, the mathematical expectation is positive for the gambling companies and therefore negative for gamblers. In other words, in the long run, the house always wins, and the more money the gambler bets, the greater the businesses’ profits will be. Ludens insists that the mathematical expectation is negative for the gambler, demonstrating this principle with examples from game rules, showing that in all games of chance operated by companies and administrations, the expected value is negative for the gambler. This is the second law of gaming, the “Expected Loss Law” or “Mathematical Hopelessness Law.”

Regarding the psychological dimension, gambling has two important repercussions in terms of health. First, it activates the same reward circuits as drugs, inducing the need to repeat the behavior (APA, 2013). That is to say, it can be addictive, which is highlighted by the third law (the “Addiction Law”). Whether a specific game is more or less addictive will depend on the characteristics of the game itself, i.e., on the rules of gambling imbedded in the game (immediacy of the reward, contingency program for reinforcement, etc.) (Parke & Griffiths, 2006), as well as the conditions under which the game occurs in society (availability, accessibility, etc.) (Williams et al., 2012) and whether these conditions favor its social expansion (advertising, marketing, etc.).

The most serious consequence of the third law is that addiction can lead to health problems whose clinical symptoms are similar to those produced by drugs: tolerance, withdrawal syndrome, difficulty in controlling one's own addictive behavior, gambling as a mechanism of escape from aversive states, etc. In other words, this is a mental disorder with diagnostic criteria similar to those for substance addictions (a list to which we should add continuing to play to recover one’s losses). These characteristics, in turn, generate a need to gamble again and, according to the second law, increase the probability of losing, aggravating the vicious cycle of gambling and loss. Pathological gamblers are those who lose more money and therefore become the gambling companies’ best clients, in keeping with the first law of gambling, the “Gambling Dynamics Law.”

Whether the concept of responsible gambling is useful for the prevention of gambling addiction is doubtful (Hancock & Smith, 2018) because, the way gambling is organized, it is a statistical certainty that some people will be affected by gambling and develop gambling disorder (Chóliz, 2018). Science is not yet able to predict which player will have a gambling disorder, but it can predict that, based on how gambling is organized in societies, a percentage of people will develop a gambling disorder. As Markham and colleagues have demonstrated, for many types of gambling, there is no threshold below which risk is fully ameliorated. For some forms of gambling, such as electronic gaming machines, every additional use increases the risk of harm (Markham et al., 2015). For this reason, the Ludens program tries to convince teenagers to reduce the frequency of gambling and, if possible, not to gamble at all.

After applying the prevention program and analyzing the results obtained in the research, the following conclusions can be drawn:

1. With regard to the secondary hypotheses (differences based on sex and age):
  The hypotheses regarding differences between males and females were corroborated. That is, males engaged in both traditional and online gambling more frequently than did females, and they also exhibited higher rates of at-risk gambling behavior and gambling disorder. These results are consistent with the extant scientific literature (Chóliz et al., 2019).
  Furthermore, as hypothesized, a greater proportion of adolescents aged 18–19 years participated in both traditional and online gambling compared to minors and engaged in at-risk gambling more frequently. No difference was found in regard to pathological gambling, suggesting that minors are more vulnerable to gambling addiction, as no differences were found in the rates of gambling disorder despite the fact that minors gambled less frequently than those aged 18–19 years. This supports the argument that it is not advisable for minors to gamble in any case.
2. Regarding the main hypothesis, i.e., that the prevention program is effective, this was confirmed in most regards, although some details should be highlighted.
  Regarding the monthly frequency of gambling, statistically significant reductions in monthly rates were found in the total sample for both traditional and online gambling. This reduction occurred in both females and males and in both minors and adolescents aged 18–19 years. These results are congruent with the main hypothesis.
  With respect to gambling problems, after the implementation of Ludens, the problems caused by gambling were significantly reduced, including both at-risk gambling (1–3 diagnostic criteria) and gambling disorder (4 or more diagnostic criteria). These results should, however, be accompanied by two remarks:
    o First, with regard to the differences between males and females, statistically significant reductions in both at-risk gambling and gambling disorder were observed among males. In the case of females, however, there was a reduction in at-risk gambling but not in gambling disorder. This may be due to the fact that the percentage of females who were initially diagnosed with gambling disorder was very low, even lower than that in a report on the Spanish population in 2015 (Chóliz et al., 2019). For this reason, the apparent reduction from 0.7% to 0.4% was not statistically significant.
    o Regarding differences by age, minors apparently benefited the most from the prevention program, as both the percentage of minors exhibiting at-risk gambling behavior and that with gambling disorder decreased significantly after the application of Ludens. These results indicate that despite the fact that minors are more vulnerable to gambling disorder, this susceptibility can be addressed with a preventive intervention such as the one presented in this article.
      However, among those aged 18–19 years, the results only showed reductions in the percentage exhibiting at-risk gambling behavior and not in that diagnosed with gambling disorder, which did not fall below a prevalence level of 3%, an excessively high level, in our opinion.
      More in-depth analysis of cases of gambling addiction in adolescents aged 18–19 years revealed that the predominant DSM-5 criterion met was preoccupation with gambling, present in 89.5% of the cases. The main games played by those over 18 with gambling disorder were casino games (78.9% of cases) and sports betting (68.4%). As it happens, advertising for casino games and sports betting has grown exponentially in Spain since the legalization of online gambling. Today, young people can go to more than 5000 gambling halls to play casino games and place bets. Advertising and marketing strategies present gambling as a leisure activity in which young people regularly participate. In fact, gambling publicity is currently one of the main topics of social and political debate in Spain.

The work we present has some limitations that must be considered. The fact that it was not possible to reduce the percentage of older adolescents with gambling disorder may be due to the fact that the addiction was already more consolidated in those individuals than in minors. In that case, a selective prevention program, or an adaptation of Ludens to newly appearing games, may be required. The Ludens program dedicated considerable time to online poker, which does not seem to be very relevant to young Spaniards. Perhaps more time should be spent on casino games and betting, as these are the games that have the highest correlation with pathological gambling in those over 18 years of age. In fact, several slides examining the most dangerous games and testimonies of young people with problems involving these games will be included in the next edition of Ludens.

Despite the difficulties and limitations, we consider Ludens to be a universal prevention program (National Institute of Drug Abuse, 1997) that has demonstrated its usefulness in reducing both the frequency of gambling and the prevalence of diagnostic symptoms for gambling disorder. The conditions under which gambling occurs depend to a great extent on the rules and regulations of the individual country. Nevertheless, the contents of the Ludens program can be easily adapted to accord with particular regulations, circumstances, and conditions, such that it can be used in different countries.

At present, in keeping with measures to restrict school entry by people outside the school system to prevent the spread of COVID-19, Ludens is being adapted so that the program can be administered by the teachers themselves in a very simple format.
